# The relevance of coagulation factor X protection of adenoviruses in human sera

**DOI:** 10.1038/gt.2016.32

**Published:** 2016-04-14

**Authors:** M R Duffy, A Doszpoly, G Turner, S A Nicklin, A H Baker

**Affiliations:** 1 Institute of Cardiovascular and Medical Sciences, BHF Glasgow Cardiovascular Research Centre, University of Glasgow, Glasgow, UK

## Abstract

Intravenous delivery of adenoviruses is the optimal route for many gene therapy applications. Once in the blood, coagulation factor X (FX) binds to the adenovirus capsid and protects the virion from natural antibody and classical complement-mediated neutralisation in mice. However, to date, no studies have examined the relevance of this FX/viral immune protective mechanism in human samples. In this study, we assessed the effects of blocking FX on adenovirus type 5 (Ad5) activity in the presence of human serum. FX prevented human IgM binding directly to the virus. In individual human sera samples (*n*=25), approximately half of those screened inhibited adenovirus transduction only when the Ad5–FX interaction was blocked, demonstrating that FX protected the virus from neutralising components in a large proportion of human sera. In contrast, the remainder of sera tested had no inhibitory effects on Ad5 transduction and FX armament was not required for effective gene transfer. In human sera in which FX had a protective role, Ad5 induced lower levels of complement activation in the presence of FX. We therefore demonstrate for the first time the importance of Ad–FX protection in human samples and highlight subject variability and species-specific differences as key considerations for adenoviral gene therapy.

## Introduction

Adenovirus (Ad), a double-stranded non-enveloped DNA virus, is the most frequently used vector in gene therapy clinical trials. The majority of the research into this large family of viruses, composed of over 65 different human types, has focused on species C Ad type 5 (Ad5). Although Ad5 has been shown to be relatively safe in the thousands of patients treated worldwide to date, efficacy following intravenous delivery has been limited. This is largely as a result of the off target host interactions endured by the virus once it comes into contact with the bloodstream. Therefore, understanding the behaviour of Ads upon exposure to blood is pivotal to their successful use in systemic gene therapy and oncolytic virotherapy applications.

Over the last decade, Ad5 interactions with host circulatory components has been the subject of extensive investigation.^1, 2, 3^ Several studies have examined the involvement of coagulation factor X (FX) in determining Ad5 tropism and immunogenicity *in vivo*.^1, 4^ Upon contact with blood, the FX Gla domain binds directly to the adenoviral hexon protein within its hypervariable regions (HVR5 and HVR7), and facilitates attachment to heparan sulphate proteoglycan receptors on the cell surface of hepatocytes.^1, 5, 6^ Although FX engagement of heparan sulphate proteoglycans has been proposed to mediate liver gene transfer, a recent study using mice lacking functional receptors has indicated that hepatocyte heparan sulphate is not essential for Ad5 liver transduction in mice.^7^ FX coating of the Ad5 hexon also has a key role in preventing the virus being recognised by murine natural antibodies, averting activation and attack by the classical complement system.^2^ Inclusion of Ad5-derived HVRs (HVR1–3 and 5–7) into a native non-FX-binding virus, Ad type 26 (Ad26), sensitised this virus to immune recognition leading to neutralisation *in vitro* and *in vivo*, and directed the virus to the liver following intravenous delivery in mice.^8^ Whilst the aforementioned studies indicate the protective benefits of the FX interaction for Ad5 infection, other work details the processes by which FX binding to the virus induces nuclear factor κB-dependant inflammatory responses following systemic vector administration in mice and heightens this arm of the host innate immunity.^9^ Evidently, FX binding to Ad5 permits important immune-modulatory functions in mice; however, dramatically less is known about these mechanisms in humans. Notably, one recent study demonstrated that in contrast to murine FX binding to Ad5 and triggering Toll-like receptor 4-mediated responses on mouse mononuclear cells, human FX was incapable of activating primary human macrophages, indicating species-specific events and highlighting the importance of using human samples for research.^10^

Major strides have been made in understanding the mediators of viral biodistribution, cellular receptor binding profiles and innate immune responses following virus intravenous administration *in vivo*. However, the majority of this work has relied heavily on small animal models, predominantly mice and unfortunately these studies can only provide limited information in terms of human translational relevance. The ability of FX to prevent Ad5 neutralisation by natural IgM antibodies and classical complement in mice has been detailed,^2, 8^ however, no data describing the significance of this pathway in humans have yet been described. In the current study, we investigate the importance of FX protection of Ad vectors from the immune components present in human sera and highlight the heterogeneous nature of the host response.

## Results and discussion

### Human FX interferes with direct binding of purified human IgM to Ad5

**Figure 1:**
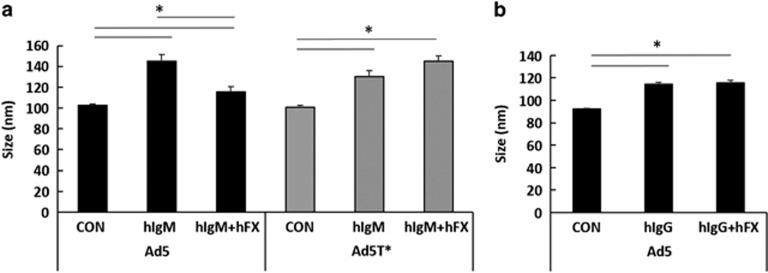
Ad5 binds hIgM in a FX-dependent manner. Alexa Fluor 532-labelled Ad5 or Ad5T* vectors (1 × 10^9^ vp) were incubated at room temperature for 90 min in phosphate-buffered saline (PBS) (10 μl final volume) with physiologically relevant concentrations of (**a**) hIgM (100 μg ml^−1^) or (**b**) human IgG (10 μg ml^−1^) in the presence or absence of human FX (5 μg ml^−1^), then diluted in 1.5 ml PBS and injected into the Nanosight. Each result is representative data from a minimum of three separate experiments with ~300 completed tracks. Mean±s.e.m. compared by one-way analysis of variance, Tukey's *post hoc* test, *P<0.05 vs matched control or hIgM conditions.

The ability of FX to bind to the Ad5 HVRs, thereby preventing recognition and attack by murine natural antibodies and complement in mice has been documented. To study the importance of this pathway in humans, we began by investigating whether human IgM (hIgM) bound to Ad5 directly and whether this was influenced by human FX. We incubated purified hIgM with Alexa Fluor 532-labelled Ad5 or the FX-binding deficient version Ad5T* (point mutations within the hexon HVR5 and HVR7^(ref. 11)^) in the absence or presence of FX and analysed alterations in virus particle (vp) size using the NanoSight LM14 (NanoSight, Malvern, UK). Binding of the free proteins to the fluorescently labelled virus correlates directly with an increase in size. The NanoSight LM14 and associated NTA software enables visualisation of nanoparticles in solution, robust particle-by-particle size measurements and quantification based on principles of Brownian motion and fluorescence light scattering.^12^ The lower size detection limit for the NanoSight LM14 is 10 nm, and whilst a hIgM pentamer is ~30 nm, FX is 9 nm and thus not detectable via this method.^12, 13^ A significant increase in particle size (~35 nm) was observed in the presence of hIgM, demonstrating a direct interaction with Ad5 (Figure 1a). Importantly, this interaction was inhibited when Ad5 was co-incubated with FX, showing the ability of FX to hinder direct binding of hIgM to Ad5. hIgM also bound the non-FX-binder Ad5T* (Figure 1a), demonstrating that the amino acid residues of the virus responsible for interacting with FX are not required for the hIgM:Ad5 interaction, and as expected, this was unaffected by FX. FX did not interfere with the binding of purified hIgG to Ad5 (Figure 1b).

### FX shielding of Ad5 and heterogeneity in human samples

**Figure 2:**
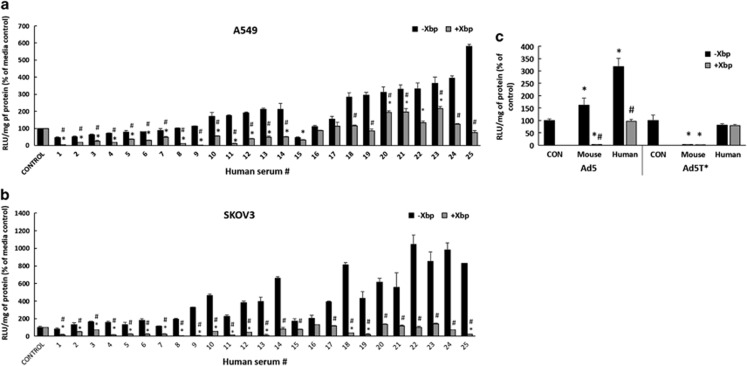
Screening human sera samples to investigate a protective role of FX. (**a**) A549 and (**b**) SKOV3 cells: Ad5 (2 × 10^10^ vp ml^−1^) were incubated with media (control) or 25 different human sera −/+40 μg ml^−1^ Xbp for 30 min at 37 °C. (**c**) SKOV3 cells: Ad5 or Ad5T* (2 × 10^10^ vp ml^−1^) was incubated with media (CON), human or mouse serum −/+ 40 μg ml^−1^ Xbp for 30 min at 37 °C. Representative human serum samples which did not show a dependence on FX for protection (pooled sera #17, 22, 24) were used in this experiment. Virus suspensions were diluted 200-fold in serum-free media and 100 μl added to cells for 2 h at 37 °C, then replaced with media with 2% fetal calf serum. Transgene expression was quantified ~16 h post transduction and relative light units (RLUs) were normalised to mg total protein. Graphs show transduction as a percentage of control (Ad transduction with media). Media control (*P<0.05) or matched serum–Xbp conditions (#*P*<0.05) vs serum+Xbp.

Next, we investigated whether hIgM and complement components neutralised Ad5 in the absence of FX in whole human sera. A panel of 25 human sera, previously identified in our lab as having low levels of pre-existing neutralising hIgG antibodies (NAbs) to Ad5 (data not shown and some serum samples identified by Parker *et al.*^14^ and renumbered for this study), was tested for effects on viral transduction *in vitro* in the absence or presence of FX. Xbp binds to the FX Gla domain and inhibits its interaction with the virus.^1^ Owing to the presence of endogenous coagulation factors in the human sera, several samples enhanced Ad5 cellular transduction, an effect significantly reduced by Xbp (Figures 2a and b). The extent to which FX enhanced Ad5 transduction varied, and this can be the result of differences in the endogenous concentrations of FX across the human subsets following blood clotting and serum production and because of altering levels of NAbs. Of the 25 sera examined, in 14 samples (56%), Xbp decreased Ad5 transgene expression to levels significantly below both media controls and serum alone (-Xbp) in A549 cells (Figure 2a). This demonstrated that without the FX protective coat, the virus is neutralised by these sera. Importantly, in the remainder of human samples (44%), Xbp did not decrease Ad5 transduction compared with controls or incubation with serum alone, demonstrating that FX was not required for basal transduction under these conditions. Similar results were observed using SKOV3 cells, although there were some differences amongst the cell lines (4 of the 25 sera caused significant neutralisation compared with media controls and serum alone in only one cell type) (Figure 2b). Previous studies in mice have shown that the ability of IgM to inhibit Ad5 gene transfer is directly related to the antibody titre, with the concentration of murine IgM negatively correlating with transduction.^15^ Variations in the levels of an individual's natural antibodies may also contribute to differences shown here amongst our human sera samples.

### Species variation in FX–Ad5 protective requirements

FX binding to Ad5 has been documented to protect the virus from immune neutralisation in mouse, guinea pig and rat sera samples *in vitro*.^2, 8^ Therefore, we performed a direct comparison between mouse sera and representative human sera (pooled sera samples #17, 22, 24; refer to Figures 2a and b) in which FX did not have a protective role. In stark contrast to mouse sera, in the absence of FX, we detected no inhibition by human sera and transduction was equivalent to the media controls (Figure 2c). Likewise, Ad5T* transduction was not affected, again in contrast to the mouse sera. Taken together, these data indicate that FX binding is not necessary to shield the virus from neutralisation in all human sera.

### FX prevents binding of sera components to Ad5 in a subset of human samples

**Figure 3:**
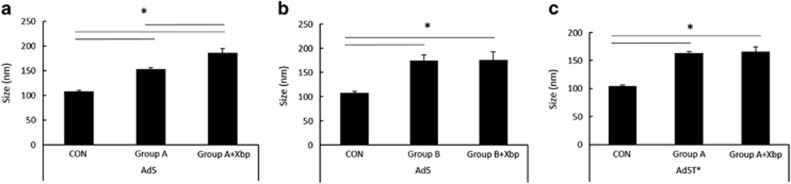
Ad binding to human serum components. Alexa Fluor 532-labelled (**a**+**b**) Ad5 or (**c**) Ad5T* (1 × 10^9^ vp) was incubated at room temperature for 90 min in human sera −/+ 40 μg ml^−1^ Xbp (25 μl final volume). (**a**) Sera Group A=sensitive to neutralisation in the absence of FX (1 × representative of this group, serum sample #5, shown here), (**b**) Group B=not sensitive to neutralisation in the absence of FX (1 × representative of this group, serum sample #24 presented here). Samples were diluted in 1.5 ml phosphate-buffered saline and injected into the Nanosight. Each result is representative data from minimum three separate experiments with ~700 completed tracks. Mean±s.e.m. compared by one-way analysis of variance, Tukey's *post hoc* test, *P<0.05 vs matched control or serum–Xbp conditions.

We next investigated whether FX coating Ad5 was preventing recognition and direct binding of components in the whole human sera, to the viral capsid. We divided our serum samples into two subsets, those in which FX was required to protect the virus from neutralisation (group A) and those in which FX protection was not necessary for cellular transduction (Figure 2). Representative samples from each subset were incubated with Ad5 or Ad5T* in the absence or presence of Xbp and vp size was measured using the NanoSight LM14. There was a significant shift in Ad5 (Figures 3a and b) and Ad5T* (Figure 3c) particle size following incubation with sera from either group indicative of interactions with serum composites. These sera represent a heterogeneous population and some possess low levels of hIgG NAbs capable of binding the virus regardless of FX (Figures 2a and b). However, only in the presence of group A sera did Xbp result in a significant increase in Ad5 particle size compared with incubation with sera alone (Figure 3a). This is indicative of binding of Ad5 by hIgM and classical complement components in the absence of FX.

### FX influences Ad5 induction of the human classical complement pathway

**Figure 4:**
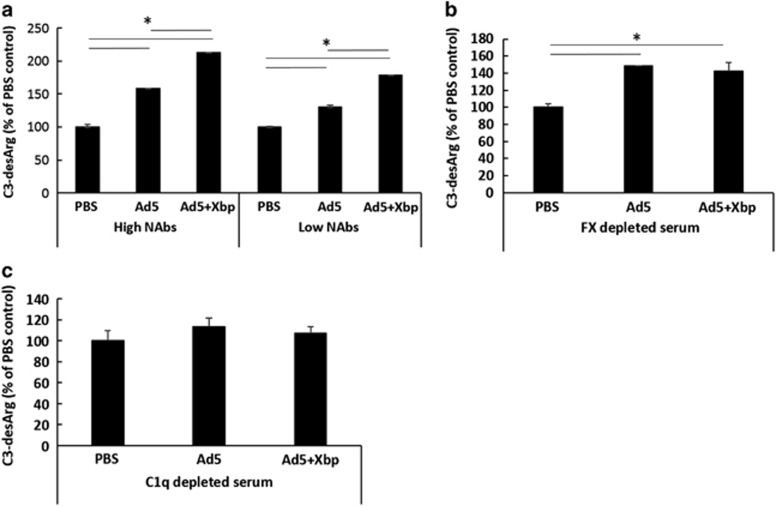
Effects on human complement activation. Human sera was incubated with Ad (5 × 10^10^ vp ml^−1^) in the presence or absence of 40 μg ml^−1^ Xbp for 90 min at 37 °C. (**a**) Human sera with high pre-existing neutralising IgG titres (high NAbs) and sera samples with low IgG titres which exhibited a dependence on FX for protection (low NAbs Group A), (**b**) FX-depleted human plasma (Quadratech Diagnostics, UK) and (**c**) C1q depleted human sera were used. C3a-desArg levels were quantified by ELISA. Graphs show transduction as a percentage of serum+phosphate-buffered saline (PBS). *P<0.05 vs matched PBS or Ad5.

We next studied the early signalling effects of hIgM binding to the virus and the activation of complement pathways. C1q is the recognition component of the classical complement cascade which can bind IgG or IgM leading to the formation of C4b2a convertase, which cleaves C3 to generate C3a.^16^ C3a is very short lived and is rapidly cleaved in serum to the more stable product C3a-desArg, hence C3a-desArg is commonly used as a measure of complement activation. We observed increased levels of C3a-desArg in both human sera with high and low pre-existing NAbs (source of serum samples Parker *et al.*^14^) following incubation with Ad5, and this was further significantly increased by adding Xbp (Figure 4a, Supplementary Figure 1). The enhanced C3-desArg signal in the absence of FX vs its presence suggests that the ability of Ad5 to induce C3-desArg is not solely reliant on FX, however, FX coating does impact upon the extent of complement-induced immunogenicity. In FX-depleted plasma, Ad5 caused comparable levels of C3a-desArg regardless of Xbp (Figure 4b). C3a and C3a-desArg are common components of classical, lectin and alternative complement pathways, whilst C1q is selectively involved in the classical system.^17^ In human serum devoid of C1q, no significant increase in C3-desArg was observed with Ad5 in the presence or absence of FX (Figure 4c). These data thereby indicate C1q-mediated C3 activation (that is, classical complement) occurs in the presence of FX, but the levels of C3a-desArg induction are significantly increased when FX is absent, in the human sera samples tested. This suggests that the human pathway is more complex than that observed in mice, in which no C3a activation occurs in the absence of FX,^2, 8^ and having no prior exposure to Ad5, the animals contain no pre-existing NAbs.

To conclude, this work shows for the first time that human FX binding to Ad5 shields the virus against neutralising human serum. However, we find there is a high level of heterogeneity amongst individual human samples in their response to Ad5 in the absence of FX, and viral coating by the coagulation factor is not always necessary for gene transfer. This is in contrast to mice, in which FX armament was essential for virus transduction in all immunocompetent models examined. In addition, we find that antibody-mediated classical complement effects on Ad5 are more complex than that previously suggested by small animal model studies, in which there is no triggering of complement pathways when FX is present. This work highlights the benefits of using translationally relevant experimental settings and human samples to investigate viral and host mechanisms. These data indicate the importance of screening patient antibody profiles prior to Ad5-mediated gene therapy, as it will be fundamental in deciphering the response to treatment.

## Materials and methods

### Cell lines and viruses

Human embryonic kidney HEK293 cells were cultured in Dulbecco modified Eagle medium (Invitrogen, Paisley, UK). Human ovarian carcinoma SKOV3 and lung carcinoma A549 cells (National Institutes of Health) were cultured in RPMI-1640 media (Invitrogen). Medium was supplemented with 2 mM l-glutamine and 10% fetal calf serum. Cells were maintained at 37 °C and 5% CO_2_. Ad5 and Ad5T* containing mutations within HVR5 (T270P and E271G) and HVR7 (I421G, T423N, E424S, L426Y and E451Q) were described previously.^11^

### Fluorescent labelling of Ad particles

Ad5 and Ad5T* were fluorescently labelled using an Alexa Fluor 532 protein labelling kit according to the manufacturer's instructions (Invitrogen). To remove any free label, the viruses were dialysed overnight using 10 000 molecular weight cutoff slide-a-lyzer cassettes (Perbio Science, Cramlington, UK) in 100 mM Tris and 50 mM EDTA. Fluorescent dye labelling efficiency was assessed using the ‘proteins and labels' function on the Nanodrop-1000 spectrophotometer (Labtech International Ltd, Uckfield, UK).

### NanoSight LM14 particle size tracking

Alexa Fluor 532-labelled Ad5 or Ad5T* vectors (1 × 10^9^ vp) were incubated at room temperature for 90 min in phosphate-buffered saline (10 μl final volume) with human serum −/+ 40 μg ml^−1^ Xbp or with physiologically relevant concentrations of purified hIgM (100 μg ml^−1^) or human IgG (10 μg ml^−1^) −/+ 5 μg ml^−1^ human FX. Purified human blood coagulation FX was purchased from Cambridge Biosciences (Cambridge, UK). Samples were then diluted in 1.5 ml phosphate-buffered saline, 1 ml of the sample was injected into the Nanosight LM14 (NanoSight) and fluorescent vp sizes were tracked. Results show representative data from a minimum of three separate experiments with ~300–700 completed tracks.

### Serum neutralisation assays

A549 and SKOV3 cells were plated in a 96-well format (1 × 10^4^ cells/well) and incubated overnight at 37 °C. Sera samples used in experiments were collected, handled and stored in the same manner. Human sera or murine sera from C57BL/6 mice were diluted to 80–90% in RPMI-1640 and incubated with 2 × 10^10^ vp ml^−1^ Ad5 or Ad5T* in a final volume of 50 μl. Xbp was added to test samples at 40 μg ml^−1^ to deplete the FX in the serum prior to the addition of virus. Controls were Ad alone in serum-free medium. Viruses were incubated with serum or medium for 30 min at 37 °C. Mixtures were diluted 200-fold in serum-free medium. Cells were rinsed and 100 μl of diluted vector (1000 vp per well) was added to triplicate wells for 2 h at 37 °C. The inoculum was then replaced with medium containing 2% heat-inactivated fetal bovine serum. After ~16 h, cells were rinsed with phosphate-buffered saline and harvested for determination of luciferase transgene expression and protein content using the BCA assay. C3a-desArg ELISAs were performed to ensure an intact complement system in a subset of sera from all groups.

### C3a-desArg ELISA

Virus (5x10^10^ vp ml^−1^) was incubated with 50 μl of human serum, FX-depleted plasma (Cambridge Bioscience) or C1q depleted serum (Quidel, San Diego, CA, USA) −/+40 μg ml^−1^ Xbp for 90 min at 37 °C, and then 10 mM EDTA was added. Samples were frozen at –80 °C until evaluation in a human C3a-desArg ELISA as previously described.^18^

### Statistical data analysis

Significance was calculated using two sample, two-tailed student's *t*-tests or where described by one-way analysis of variance with Tukey's *post hoc* test. *P*-values of <0.05 were considered to be significant. Results presented are representative data from a minimum of three separate experiments with at least three experimental replicates per group. All error bars represent s.e.m.
