# Supplementary figures and images for: The relevance of coagulation factor X protection of adenoviruses in human sera
Source: Gene Ther. 2016 Apr 14;23(7):592–6. doi: 10.1038/gt.2016.32 (PMC4940928; doi:10.1038/gt.2016.32)

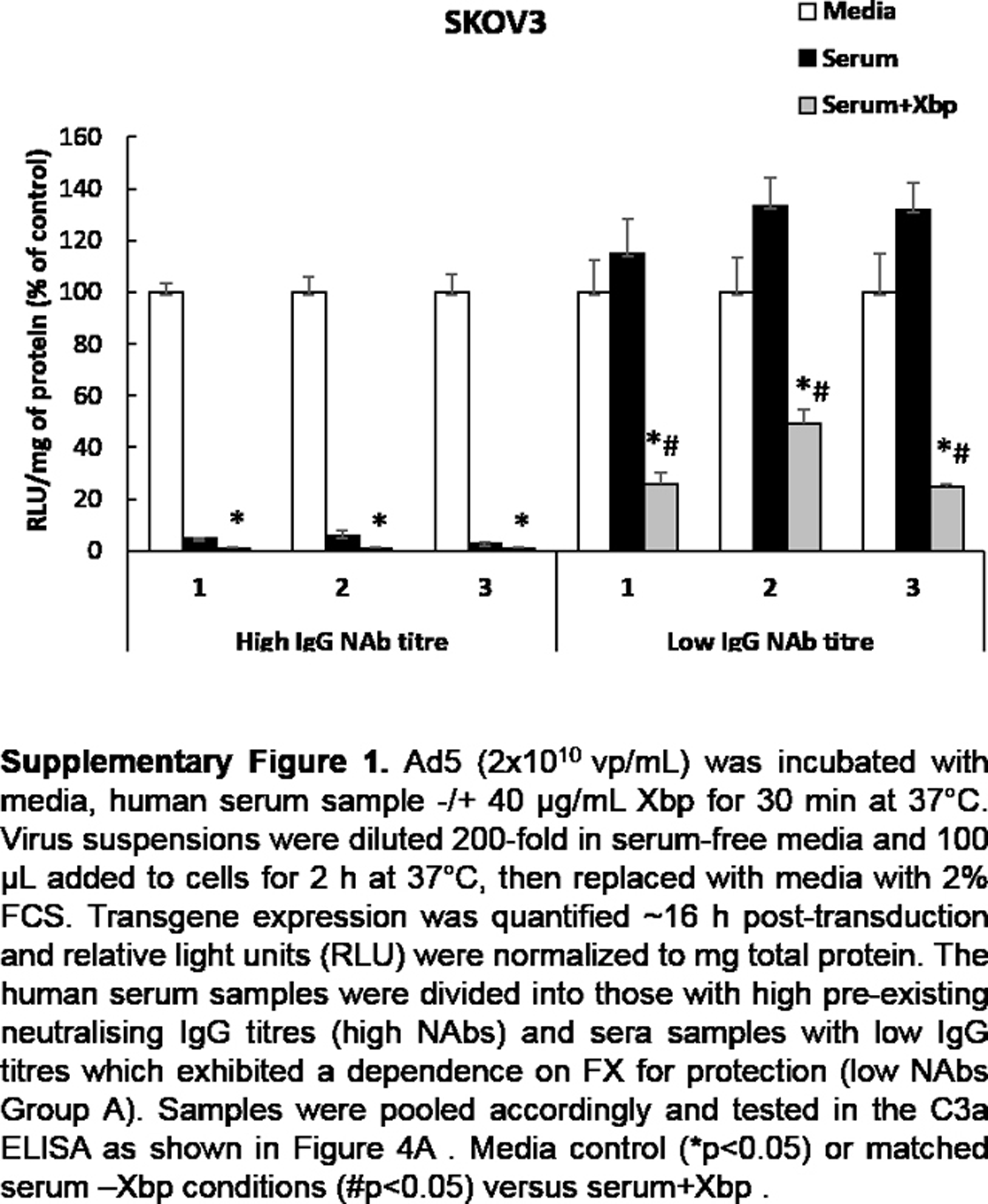

Supplement: Supplementary Figure 1 [file gt201632x1.tif]
